# The growth of capillary networks by branching for maximum fluid access

**DOI:** 10.1038/s41598-023-38381-6

**Published:** 2023-07-12

**Authors:** Xuewei Zhang, Sylvie Lorente

**Affiliations:** Mechanical Engineering Department, Villanova University, 800 Lancaster Ave., Villanova, PA 19085 USA

**Keywords:** Engineering, Mechanical engineering

## Abstract

Here we document the deterministic evolution of capillary networks that morph by connecting more and more branches to water sources. The network grows with the objective of extracting in steady state higher and higher liquid flow rates. Growth happens through the generation of tree-shaped structures and the geometrical configuration of the dendritic network evolves as the number of connected sources increases. We present a novel methodology to generate capillary architectures and show how the evolution of the network leads to pump higher volumetric flow rates by capillary suction. The results suggest that networks generated within a plane lead to higher flow rates than networks generated within a three-dimensional domain, for the same volume of fluid.

## Introduction

Capillary flows, the movement of liquid under the driving force generated at the interface between liquid and gas in confined space, are ubiquitous in engineering, with applications in a range of domains covering construction and civil engineering^1^, microfluidics^2^, or aeronautics^3^. For example, Lee et al.^2^ studied spontaneous capillary flow in uniform cross-section open-channel capillary grooves for applications in space science, biological and chemical analysis. Flow behavior at a bifurcation was investigated and well-dimensioned bifurcations are proven to increase the flow rate in the capillary pump. Huang et al.^4^ designed and manufactured a porous metal module with biomimetic tree-like micro channels for self-pumping and self-adaptive transpiration cooling. The self-pumping porous module efficiently cools down the heated surface and automatically adjusted the coolant mass flow rate to encounter the changed heat flux while maintaining the approximately same cooling performance. The porous module can be used to protect the leading edge of hypersonic spacecraft from the tremendous aerodynamic heating. Liu et al.^5^ introduced a framework to achieve a controllable flow velocity by tailoring the cross-sectional profile of homogeneous porous media. A fluidic device with controllable capillary flow process in porous media was achieved by assembling the designed capillary elements which can provide broad flow adjustability for paper-based microfluidic devices. It is worth noticing that such hierarchical fluidic networks can be built at very small scales with today's technology^6–9^.

In the field of construction materials, permeable pavements are viewed as a promising solution to alleviate the urban heat islands phenomenon through evaporation of capillary water at the surface of the materials^1^. Liu et al.^10^ reported that the capillary capacity of permeable pavements has a positive correlation with the evaporation rate and temperature drop.

In all these domains, controlling the amount of water that the network can extract by capillary suction is of paramount importance. Access to water in an efficient way is of the same importance in natural systems. For example, plants root branching occurs with the objective of maximizing water uptake, as in hydrotropism^11^. In Ref.^12^, the authors consider that plants spread their roots in all directions but can detect where water (and nutrients) is located. The root network remains always saturated with liquid (mixture of water and nutrients) while actively growing branches towards water sources^13^. Such behavior leads to the generation of complex dendritic networks, where complexity must be understood as root architectures with large branching levels spreading throughout the available space.

The commonality between those flow systems, whether natural or man-made, is that their architecture is morphing with a specific purpose: the one of increasing flow access. The evolution of flow configurations happens as a result of the reduction in flow resistances through changes in channels diameters and lengths, and through branching, with a constant fluid volume. The idea of networks morphing to facilitate access to the currents that flow through them is encapsulated in the constructal law of evolutionary design^14,15^ for both engineering and natural systems. Manifestations of the constructal law are numerous in nature, societal^16–18^ and engineering applications^19–22^. The fundamental problem of how fluid networks grow to passively pump water, i.e. thanks to capillary suction, belongs to the constructal field.

This work proposes to uncover how the growth of a water capillary network can be predicted by allowing the network to choose its branching among randomly distributed sources, for maximum flow rate. To this sake, we propose a theoretical approach showing how the evolutionary network morphs entirely its structure to extract the highest flow rate possible while maintaining enough capillary strength to overcome the global friction losses. To show the capillary networks growth, we develop an in-house numerical tool inspired by works describing blood flow circulation in the human body. The CCO (Constrained Constructive Optimization) algorithm was originally proposed by Schreiner and Buxbaum^23^, and is widely used for the construction of binary vascular trees in the field of health sciences. The algorithm allows to model blood flow through arterial trees based on Poiseuille law. Developments followed to describe the blood vasculature of the kidney^24^, the liver^25,26^, and the inner retinal vascularization^27^. The CCO starts with sources randomly placed, and connects the sources to the network one by one. At every step of adding a new source to the network, geometrical constraints are applied, and the geometrical features of the channels are modified to minimize the energy required for the fluid to flow through the network. Keeping the initial features of the CCO approach^23^, we develop a completely different algorithm dedicated to capillary flows in which the network grows by letting new channels spring out to connect to new water sources for higher flow rates, while morphing its configuration for minimum global flow resistances.

We are not interested here in the transient behaviors. We consider the network growth for maximum fluid access once in steady state. This means that among the various branching possibilities, the one kept is the one leading to the highest flow rate at the network outlet once the entire system is in steady state.

## Results

We consider a domain (disk in 2D, sphere in 3D) which contains $N$ water sources randomly generated. The outlet of the growing capillary network is located on the periphery of the domain. The capillary network is made of cylindrical channels, and it grows by adding new cylindrical branches to connect one water source after the other. Growth is happening by dichotomy. The ratio between a parent duct and the children channels follows Hess-Murray law^28–31^ in the first part of the work, before its impact on the flow rate is studied later on. The detailed assumptions are presented in the “Model” section.

**Figure 1:**
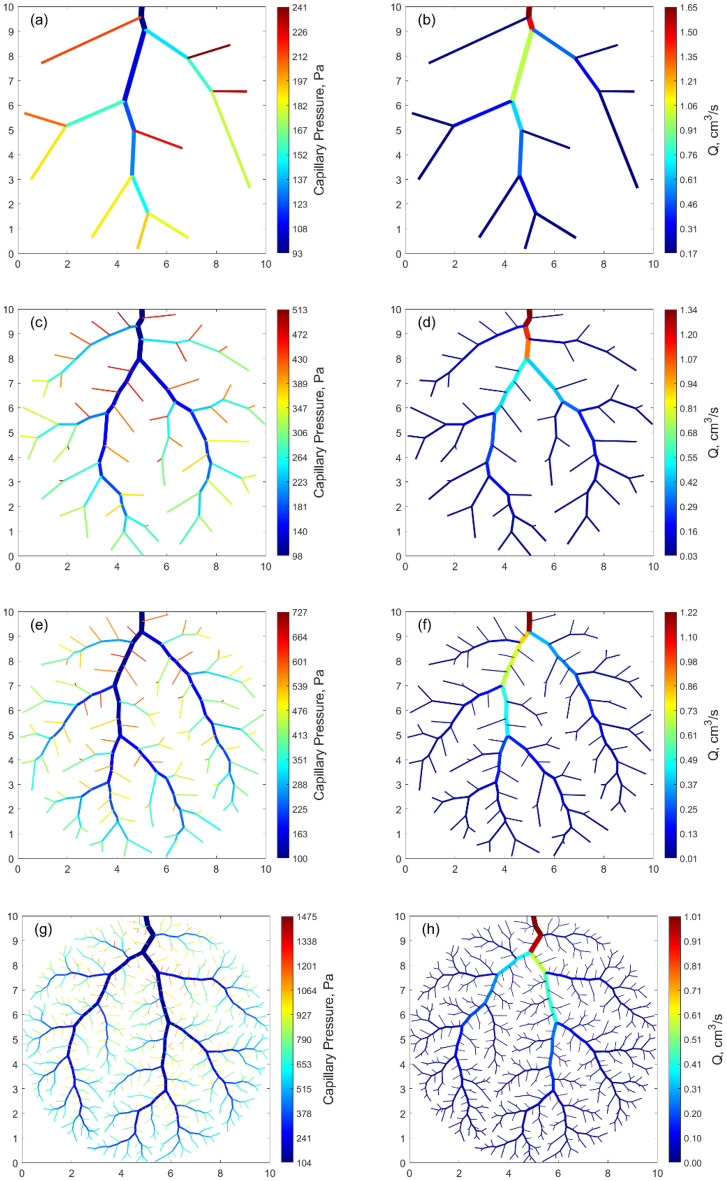
Capillary pressure (left) and flow rate (right) distribution at the end of the network generation in a circular domain. (**a**) and (**b**) $N=$ 10 inlets, (**c**) and (**d**) $N=$ 50 inlets, (**e**) and (**f**) $N=$ 100 inlets, (**g**) and (**h**) $N=$ 500 inlets.

Figure 1 exhibits the capillary pressure within the network at the end of the growth, together with the flow rate distribution. In the figures, the thickness of the channels is proportional to the actual diameter of the ducts. The networks are made of $N=$ 10, 50, 100, 500 inlets. They are generated within a circular domain of identical radius. The movie of invasion phase is provided in the supplementary material (Movie S1–S4) for $N=$ 100 and $N=$ 500.

The network grows new channels to connect new sources, and at every growth step the total volume of the network adds a new elemental volume $\delta V$. From one step of growth to the next one ($i$ inlets to $i+1$ inlets), the overall volume gain is $\delta V$. This does not mean that $\delta V$ is added to the newly created branch: $\delta V$ is distributed through the network modifying diameters and length of the channels in such a way that the flow rate at the outlet is maximum while being equally distributed among all the inlets.

**Figure 2:**
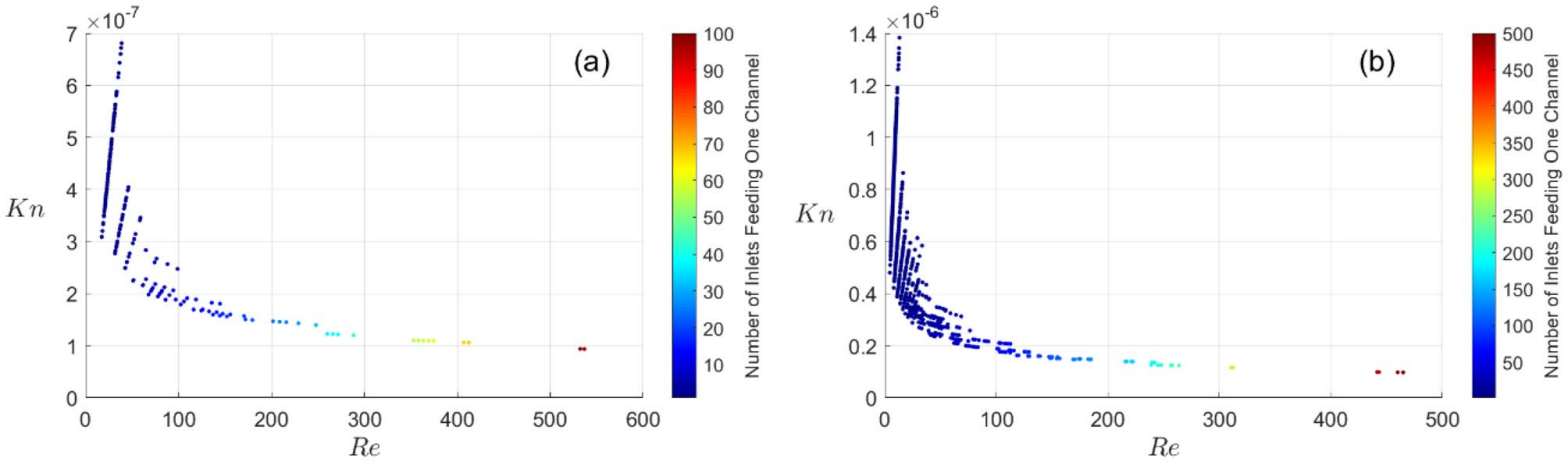
The Knudsen number in every channel of a network of $N=$ 100 (**a**) and $N=$ 500 (**b**) inlets. The capillary network is generated within a circular domain.

The approach developed in this work relies on the assumption that the flow through the capillary remains laminar from inlet to outlet (see Eq. (1) in the ‘Model’ section). The assumption of continuous flow is verified when the Knudsen number ${K}_{n}={\lambda }_{water}/2r$ remains below 0.01 in all the channels, where ${\lambda }_{water}$ is the water mean free path length ($\sim 2.7$ Å). We show in Fig. 2 an example of result in the case of networks generated in circular domains with $N=$ 100 and $N=$ 500 inlets. The final volume of the network is the same in both cases ($1 c{m}^{3}$). The Knudsen number was calculated in every channel, together with the corresponding local Reynolds number, $R{e}_{i}=2{r}_{i}{U}_{i}/\nu$, where the average velocity ${U}_{i}$ is given by ${Q}_{i}=\pi {r}_{i}^{2}{U}_{i}$. Figure 2 must be understood as a photography of the flow network characteristics at the end of the network generation. For example, when $N=$ 500 (Fig. 2b), the channel receiving the fluid from $N=$ 500 sources is located on the right-hand side of the graph with a Reynolds number of 465, and a Knudsen number of ${10}^{-7}$. This channel is the network outlet. Moving to the left of the graph, we see that the number of capillaries fed by fewer sources increases. They correspond to smaller Reynolds numbers and higher Knudsen numbers. Nevertheless, the flow remains continuous in all the channels.

**Figure 3:**
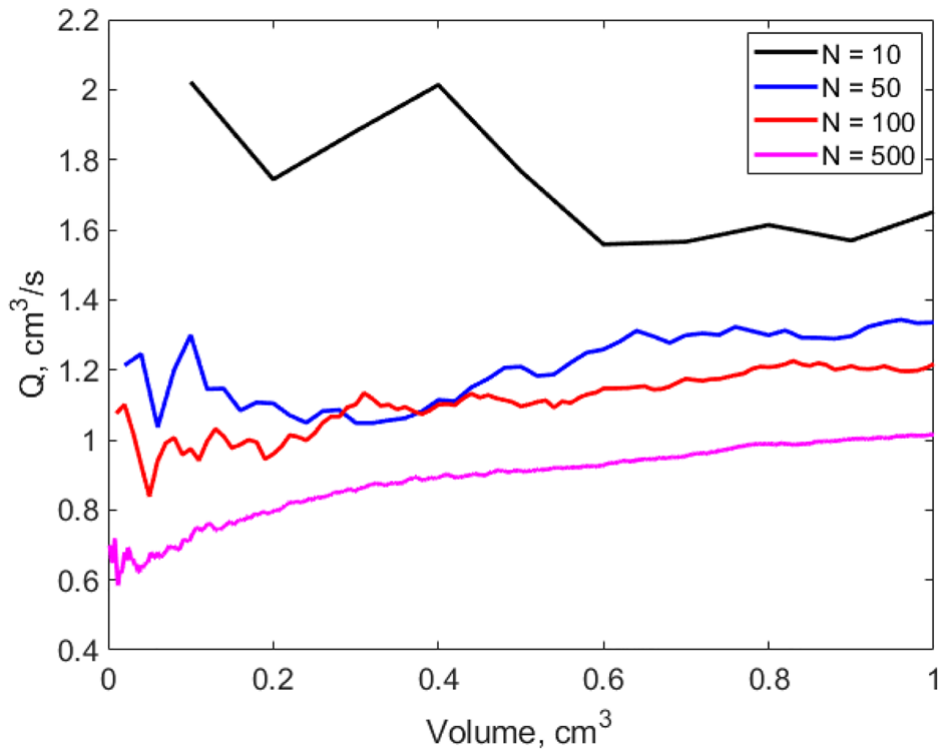
Volumetric flow rate for increasing network volume when $N=$ 10, 50, 100 and 500 inlets in a circular domain.

Plotted in Fig. 3 is the volumetric flow rate reaching the outlet of the capillary network as a function of the network volume increase. During growth, the extracted flow rate increases, except for $N=$ 10 inlets. The balance between the outlet channel capillary pressure and the friction losses along any fluid pathway must be fulfilled, while the capillary strength $C{S}_{i}$ (defined later in Eq. (7)) remains positive everywhere, and the radius of the channels obeys Eq. (3) at every bifurcation. This leads to flow rates decreasing locally, especially at the start of the network growth. Beyond this initial phase, the volumetric flow rate continuously increases. Figure 3 indicates that the flow rate obtained at the end of the network evolution is smaller for high numbers of inlets. Connecting a high number of inlets in a territory of identical size requires many thinner channels. Even though the capillary pressure increases (as a function of ${r}^{-1}$, $r$ being the channel radius), the friction losses increase much faster (as a function of ${r}^{-4}$). For example, moving from a capillary network made of $N=$ 50 inlets to a network of $N=$ 500 inlets leads to a flow rate decrease of 21.5%.

**Figure 4:**
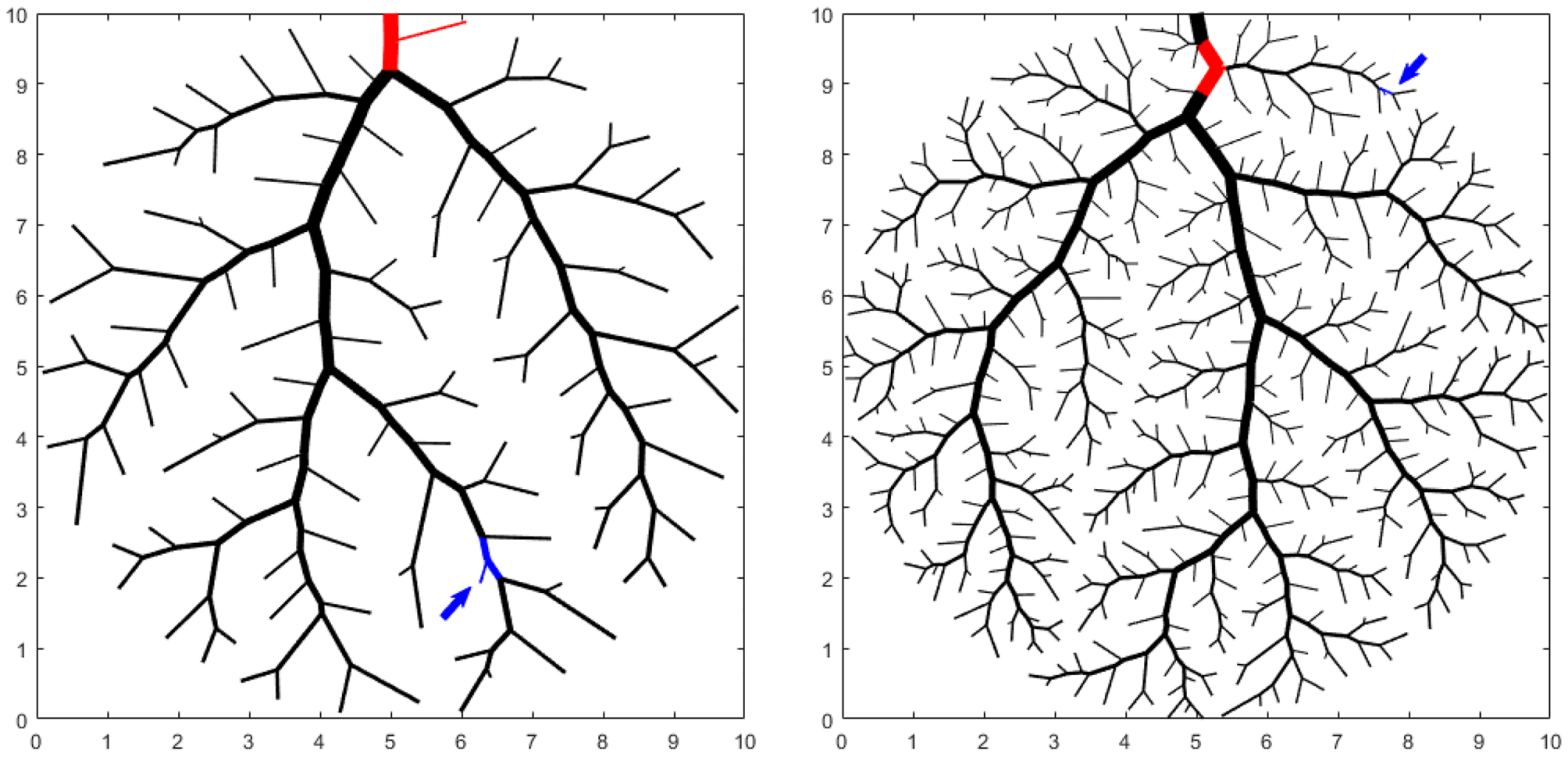
Location of the maximum angle of bifurcation (in red) and the minimum one (in blue) when $N=$ 100 (left) and $N=$ 500 (right).

We calculated the average angle of bifurcation $\beta$ for every network and obtained $\beta =71.5\pm 0.5^\circ$. Interestingly, the value of the bifurcation angle is very close to the predicted angle when a dendritic network is simply made of a parent tube and two children channels, and this Y-shaped network is inscribed in a circular domain^32^. In such a case, a fully developed laminar stream flows through the network and the lowest pressure losses are obtained when the radius ratio obeys Hess-Murray’s law and the bifurcation angle is 75$^\circ$. A closer look at the results indicates that the minimum and maximum angles of bifurcation within the network oscillate between 45$^\circ$ and 95$^\circ$ when $N=$ 10, between 30$^\circ$ and 128$^\circ$ when $N=$ 50, 32$^\circ$ and 125$^\circ$ when $N=$ 100, and 31$^\circ$ and 128$^\circ$ when $N=$ 500. For the sake of illustration, Fig. 4 shows the location of the minimum and maximum angles of bifurcation when $N=$ 100 and $N=$ 500. Note that the angles with minimum value are in the vicinity of the sources while the angles with maximum value correspond to bifurcations on the largest vessels close to the outlet of the capillary network.

**Figure 5:**
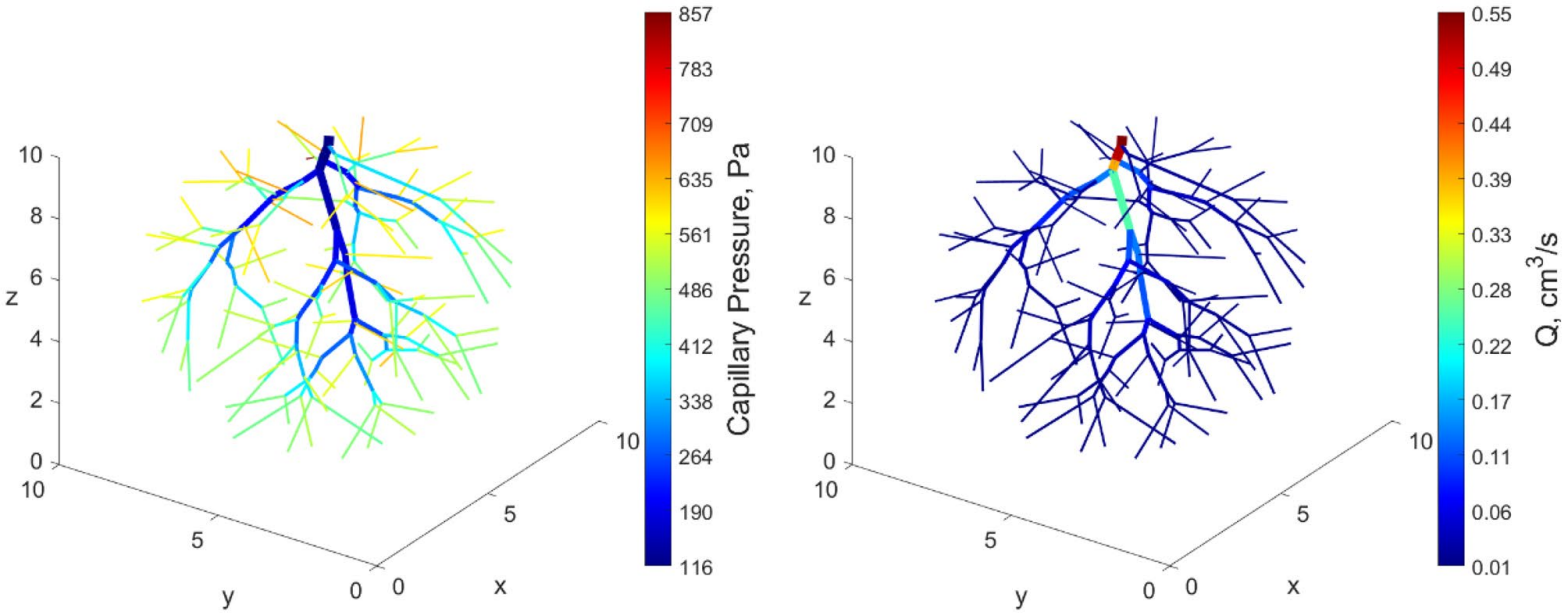
An example of 3D capillary network with $N=$ 100 inlets. The capillary pressure (left) and flow rate (right) distribution are shown at the end of the generation.

The capillary networks were also grown in 3D, replacing the circular domain by a spherical one. An example of the result is shown in Fig. 5 (end of growth) and in the movie of the supplementary material (Movie S5, S6) when $N =$ 100. The total network volume at the end of the growth remains $V=1 {\mathrm{cm}}^{3}$.

## Liberating the configuration

**Figure 6:**
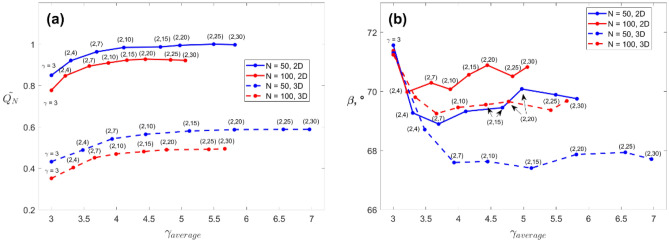
(**a**) Non-dimensional flow rate $\widetilde{{Q}_{N}}$, and (**b**) bifurcation angle $\beta$ as a function of the average bifurcation ratio ${\gamma }_{average}$. The results are compared between different ranges of $\gamma$ in the networks.

So far the capillary networks grew by assuming that their geometry is in accord with the diameter ratio corresponding to Hess-Murray’s law (Eq. (3)). Yet, our previous work^33^ showed that in capillary networks the exponent $\gamma$ in Eq. (3) diverges from the value 3 when the channels are in the vicinity of the network outlet. Therefore, the capillary networks were generated again, both in 2D and 3D, by considering that the relationship between the diameter of a parent and two children channels is a degree of freedom in the change in network morphology. This is obtained by allowing the diameter ratio at each splitting level to vary within a certain range. The results are plotted in Fig. 6. They present the flow rate exiting the capillary network at the end of growth for $N=$ 50 and $N=$ 100. The flow rate is given in a non-dimensional way ($\widetilde{{Q}_{N}}$) by dividing the obtained flow rate at the end of the growth (for one given configuration) by a maximum value. The latter corresponds to the flow rate of a network generated in a circular plane with $N =$ 50 inlets when the exponent $\gamma$ varies in the range (2, 25). The values are provided as a function of the exponent ratio ${\gamma }_{average}$. Note that the value of ${\gamma }_{average}$ used to build the two graphs is the average over all the network bifurcations and corresponds to the average over more than 15 network generation tests. The range of values between which $\gamma$ could vary during the capillary network growth is provided between parentheses. For example, for a 3D network made of $N=$ 100 sources when the diameter ratio can vary between $\gamma =2$ and $\gamma =10$, the ${\gamma }_{average}$ value that allows the maximum flow rate leaving the network is ${\gamma }_{average}=4$, corresponding to a non-dimensional flow rate $\widetilde{{Q}_{N}}=$ 0.47. We note that the networks generated on a plane (2D configurations) always lead to higher flow rate extracted compared to networks growing in a volumetric space. The ratio between the obtained flow rates is about 2. Connecting the network to sources distributed within a circular plane or a spherical volume, both having the same diameter, means that the channel lengths must be longer and thinner in the sphere domain than in the plane one. This leads to higher friction losses, and, consequently, to lower flow rates at constant fluid volume.

When given the freedom to choose the way the branching vessels connect to the parent ducts, the ${\gamma }_{average}$ value for maximum flow rate remains close to the lowest boundary of the allowed range. Furthermore, the change in the exponent appears to have little impact on the flow rate reached at the outlet, as Fig. 6a shows that all the curves tend to reach an asymptote. By the same token, the corresponding average angle of bifurcation becomes also relatively insensitive to the change in $\gamma$.

**Figure 7:**
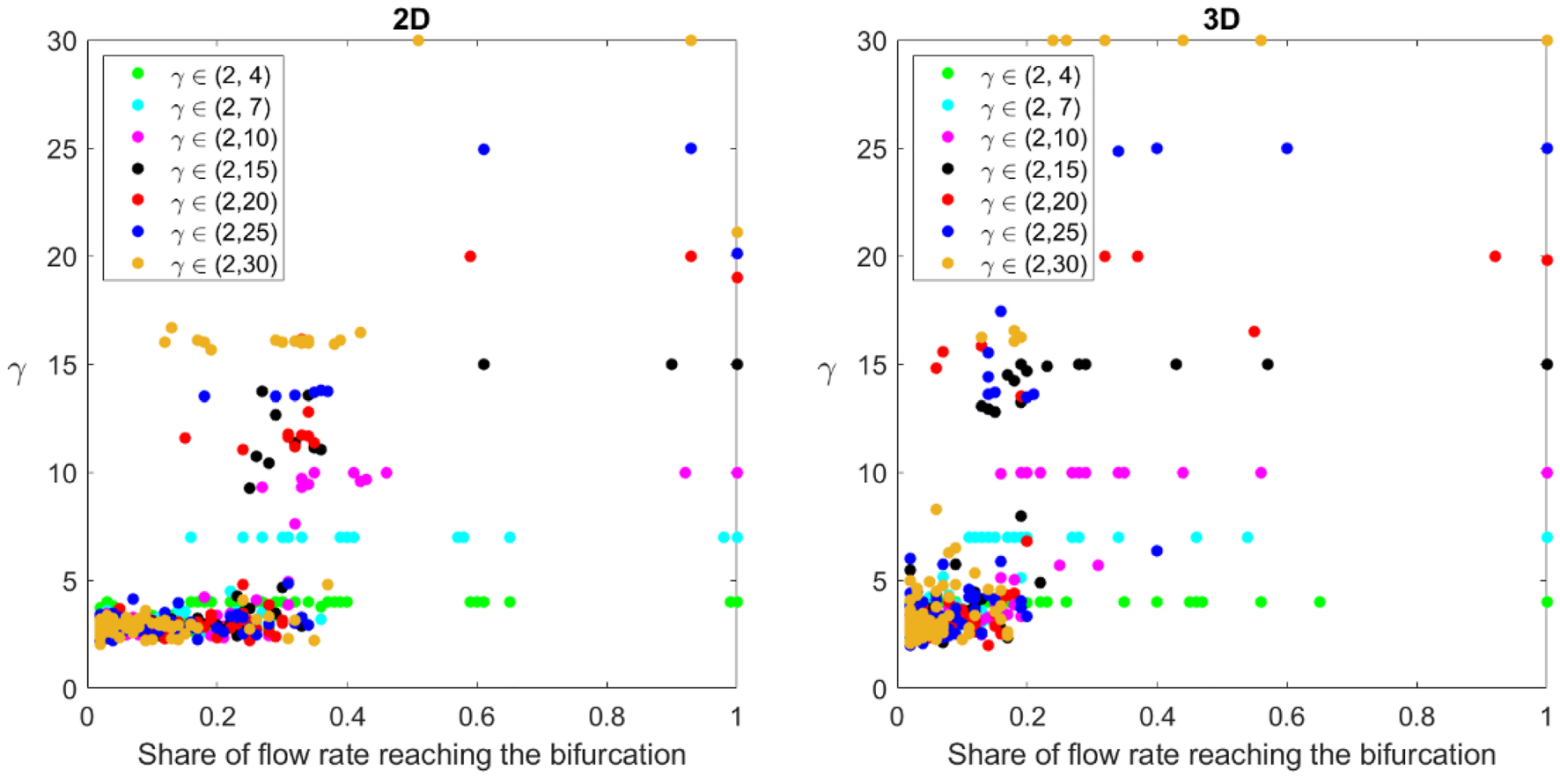
Local distribution of bifurcation ratio $\gamma$ for both 2D (left) and 3D (right) networks with 100 inlets. The results are compared between different ranges of $\gamma$ in the networks.

Figure 7 presents the local value of $\gamma$ in a 2D and a 3D network made of $N=$ 100 inlets. The figure is constructed to picture the distribution of the $\gamma$ values for bifurcations over the entire network. One data point represents the bifurcation that receives the flow rate from a certain percentage of the sources. The more we move to the right-hand side of the graph, the closer to the outlet of the network. Take the 2D network as an example, when $\gamma$ is allowed to vary in the range of (2, 25), the highest values of the exponent are obtained at the three bifurcations receiving respectively 61%, 93%, and 100% of the total inlets flow rate. It means that the parent channel radius is very close to the children’s channels one. Then a group of 6 bifurcations continues to have a high $\gamma$ while most of the bifurcation points see a more significant change in the radius ratio with the exponent Eq. (3), $\gamma \widetilde{=}3$. This case corresponds to the bifurcations receiving up to about 40% of the flow.

A similar analysis can be conducted for the three-dimensional case (graph on the right in Fig. 7). Keeping the example of $\gamma$ allowed to vary between 2 and 25, we see that the group of bifurcations with a significant change in diameter from parent to children ($\gamma <5$) receives the fluid from a lower number of inlets. In sum, the network follows the Hess-Murray law away from the outlet.

## Model

The network is made of an assembly of cylindrical capillary tubes of radius $r$ and length $l$. Gravity effect is neglected due to the small dimensional of the system. The flow rate entering the network is identical for every inlet. Yet its value changes along the construction of the network. We consider steady state and therefore assume that the entire volume of the capillary network is filled with the liquid. To maintain the flow rate $Q$ at the end of a channel of radius $r$ and length $l$, the balance between capillary pressure and friction losses requires

1 $$\frac{2\sigma cos\theta }{r}=\frac{8\mu }{\pi }\frac{Ql}{{r}^{4}},$$

where $\sigma$ is the surface tension (0.072 $N/m$ for water/air), $\theta$ is the contact angle ($\theta =0^\circ$ in accord with^34^), $\mu$ is the dynamic viscosity (${10}^{-3} Pa\cdot s$ for water). The left-hand side of Eq. (1), known as the Laplace equation, gives the capillary pressure. The right-hand side of the equation assumes that the flow remains in the laminar regime. We also assume and verify later that the Knudsen number is lower than 0.01 in every duct (see Fig. 2). Note that the Bond number was calculated ($Bo=\rho g{L}^{2}/\sigma$, with L the length of the channel connected to the outlet) and was always below 1, meaning that the surface tension forces dominate the flow and therefore justifying neglecting the effect of gravity.

Along a flow pathway, from any inlet to the network outlet, the equivalent relation to Eq. (1) for the entire network is given by

2 $$\frac{2\sigma cos\theta }{{r}_{outlet}}=\frac{8\mu }{\pi }\sum_{i}{Q}_{i}\frac{{l}_{i}}{{r}_{i}^{4}}.$$

An additional constraint to allow realistic geometries is that ${l}_{i}/{r}_{i}>2.$ The capillary network grows by branching in such a way that the radii of the three branches that constitute a dendrite are related to each other by

3 $${r}_{p}^{\gamma }={r}_{c1}^{\gamma }+{r}_{c2}^{\gamma },$$

where *p* stands for parent, and *c1*, *c2* mean child 1 and child 2.

In flows driven by a pressure difference, such as the blood flow in the animal body, Hess^28,29^ and Murray^30,31^ demonstrated that the exponent $\gamma$ should be 3 to work with minimum fluid volume for a constant flow rate. The law assumes that the flow is laminar. It famously describes with good accuracy fluid transport in the animal respiratory system together with the vascular system. We showed recently^35^ that searching for minimum fluid volume at constant flow rate is identical to searching for maximum flow rate at fixed volume. This is the approach taken in this work.

For example, in the case of three branches, the flow rate will be obtained from Eq. (1), the overall pressure losses balance (Eq. (4)) and flow rate conservation (Eq. (5))

4 $${Q}_{c1}{l}_{c1}/{r}_{c1}^{4}={Q}_{c2}{l}_{c2}/{r}_{c2}^{4},$$

5 $${Q}_{c1}+{Q}_{c2}={Q}_{p},$$

leading to

6 $${Q}_{p}=\frac{\pi }{4\mu }\frac{\sigma cos\theta }{{r}_{p}}{\left[\frac{{l}_{c1}/{r}_{c1}^{4}}{1+{\left({r}_{c2}/{r}_{c1}\right)}^{4}{l}_{c2}/{l}_{c1}}+\frac{{l}_{p}}{{r}_{p}^{4}}\right]}^{-1}.$$

Equation (6) must also satisfy Eq. (3) and the constant fluid volume requirement, $\sum_{i}\pi {r}_{i}^{4}{l}_{i}$, at any given step of growth. The location of the connection between the three channels and the radius ratio is the parameter that allows to obtain the highest flow rate.

**Figure 8:**
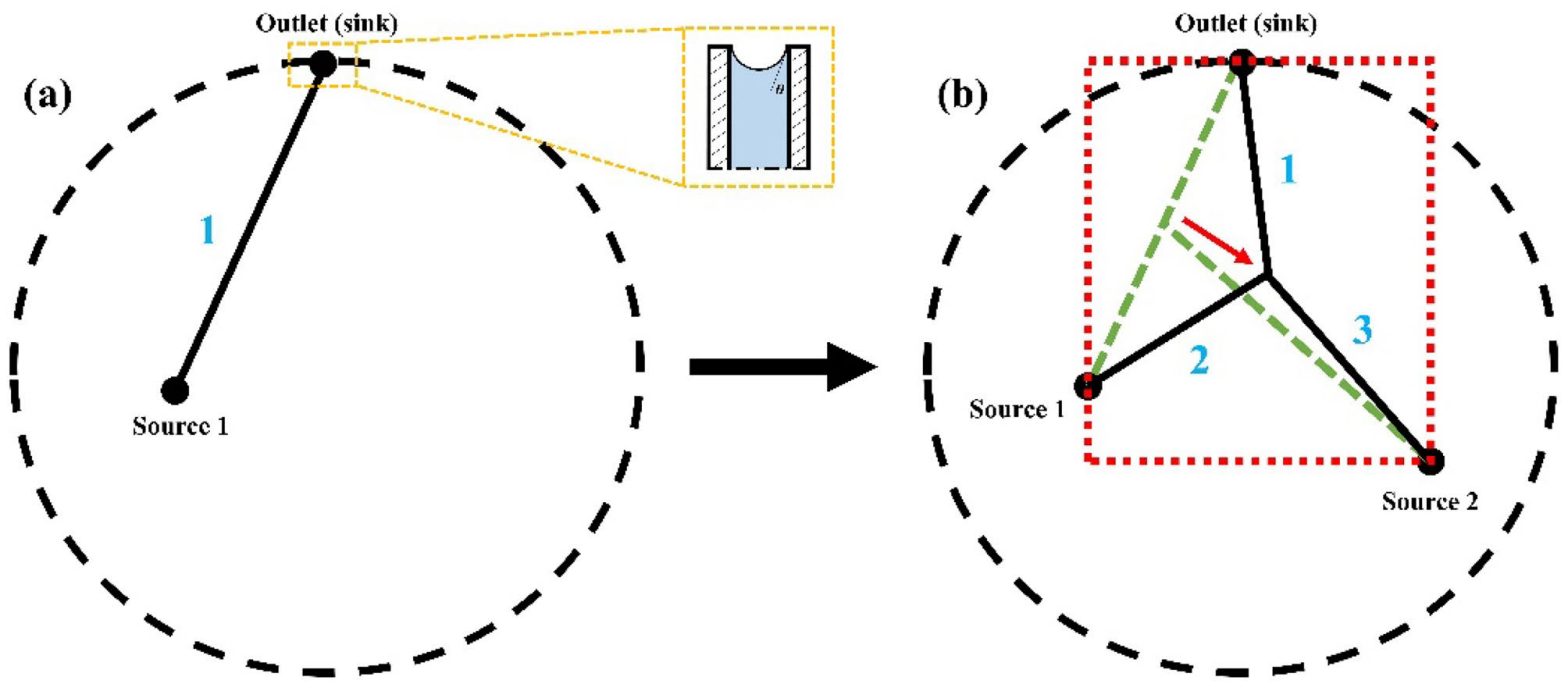
(**a**) The network starts with channel 1 of radius ${r}_{1}$ linking an inlet (source 1) to the outlet (sink). (**b**) Source 2 is randomly located within the domain bounded by dotted circle. Channel 3 springs out from the mid-length of channel 1 connecting source 2. Channel 1 is split into channels 1 and 2, resulting in a network of three channels (the initial network is shown in green dotted lines). The connection location is then moved within the red dashed red box, and radius and length changed at constant volume, until the maximum flow rate (steady state) is obtained at the outlet of the network (black solid lines in (**b**)).

We need to ensure that the driving force (i.e. capillary pressure) in every channel is strong enough to overcome the friction losses due to the flow rate in the channel that leads to the maximum flow rate at the outlet. Calling Capillary Strength (CS) the difference between the capillary pressure in a channel $i$ and the friction losses upstream the outlet cross-section of the same duct, we write

7 $$C{S}_{i}=\frac{2\sigma cos\theta }{{r}_{i}}-\frac{8\mu }{\pi } \sum_{j=1}^{i}{Q}_{max,j}\frac{{l}_{j}}{{r}_{i}^{4}},$$

where ${Q}_{max,j}$ is the flow rate in a channel $j$ such that the flow rate at the entire network outlet is maximum. The three-branch case is shown in the network of Fig. 8, which connects two sources to one sink. The three-branch network is filled with water which flows in laminar regime thanks to capillary suction. The interface between water and the air is also shown in Fig. 8.

## Network generation

While the capillary network grows, so does the domain around it. In a two-dimensional configuration, to enlarge the domain as the network generation proceeds, the radius of the domain, ${R}_{i}$ is calculated as

8 $$\pi {R}_{i}^{2}=\left(\frac{i}{N}\right)\pi {R}_{N}^{2},$$

where $i$ is the current inlet number and $N$ is the total inlet number. Here, the radius of the network domain ${R}_{N}$ is 5 cm, when $N$ inlets are connected to the network.

The first channel of volume $\updelta V$ is created by connecting a first inlet, randomly placed, to the outlet. The outlet location is chosen on the perimeter of the domain. The first channel is then scaled up to a volume of $2\updelta V$ as the radius of the domain is increased from ${R}_{1}$ to ${R}_{2}$. This allows to maintain the ratio, volume network/domain area constant, before adding new branches. Next, a random source is located in the ${R}_{2}$ domain, and a new channel springs out from the mid-length of the initial duct, and connects to the source. This splits the first channel into two. By varying the connecting point and the radius of the channels for maximum flow access, while obeying Hess-Murray’s law and keeping the network volume constraint at 2 $\updelta V$, the final location of the connection is obtained (Fig. 8). During the process, the connecting point is moved within the rectangular area where the three involved channels reside (red dotted rectangle in Fig. 8b). The connecting point will be located in the triangle delineated by source 1, source 2, and the outlet of the mother channel for best flow performance. The network grows by tentatively connecting to a new source and the new channels springing out from the 20 ducts located in the closest vicinity of the new source. These 20 possibilities are each tested by reconfiguring the network for maximum flow access at constant volume $\sum_{1}^{i}\delta V$, as just described. The case leading to the maximum flow rate is kept, out of the 20 possibilities, before continuing to grow and morph the network following the same methodology. As branching modifies the pressure equilibrium within the network, the vasculature geometry changes within the entire flow architecture to ensure that the capillary strength overcomes the friction losses along every fluid pathway at constant overall fluid volume.

An in-house code was developed in MATLAB^36^. Networks were created in 2D and in 3D, replacing the circular domain with a spherical one. In each case, a network was generated more than 15 times before collecting the average flow rate. Indeed, our previous work^35^ showed that the generation of about 15 random networks for a given number of inlets was sufficiently representative.

## Concluding remarks

In this work, we developed capillary networks that spread and morph by generating new branches to connect an increasing number of random water sources. We showed that the directional growth of the network can be predicted for extracting by capillarity the maximum flow rate possible. When new branches develop from the existing network, the preferred one allows to increase the overall flow rate while reconfiguring the network for lower friction losses. The flow configuration morphs by changing the geometrical features at constant total volume to ensure that the capillary strength is high enough to balance the additional friction losses.

Finally, for the same total fluid volume and number of sources, we show that a network generated within a plane extracts twice as much water than when generated within a volume.

## Supplementary Information

Supplementary Legends.

Supplementary Video S1.

Supplementary Video S2.

Supplementary Video S3.

Supplementary Video S4.

Supplementary Video S5.

Supplementary Video S6.

## Data Availability

The datasets used and analyzed during the current study available from the corresponding author on reasonable request.
