# Supplementary material for: The growth of capillary networks by branching for maximum fluid access
Source: Sci Rep. 2023 Jul 12;13:11278. doi: 10.1038/s41598-023-38381-6 (PMC10338522; doi:10.1038/s41598-023-38381-6)
Supplement: Supplementary file 1 — Supplementary Legends. [file 41598_2023_38381_MOESM1_ESM.docx]

Supplementary Information Guide

Supplementary Movie S1 Movie of the generation of a 2D network with 100 inlets. The flow network is colored to reflect the non-dimensional capillary pressure in each channel.

Supplementary Movie S2 Movie of the generation of a 2D network with 100 inlets. The flow network is colored to reflect the non-dimensional flow rate in each channel.

Supplementary Movie S3 Movie of the generation of a 2D network with 500 inlets. The flow network is colored to reflect the non-dimensional capillary pressure in each channel.

Supplementary Movie S4 Movie of the generation of a 2D network with 500 inlets. The flow network is colored to reflect the non-dimensional flow rate in each channel.

Supplementary Movie S5 Movie of the generation of a 3D network with 100 inlets. The flow network is colored to reflect the non-dimensional capillary pressure in each channel.

Supplementary Movie S6 Movie of the generation of a 3D network with 100 inlets. The flow network is colored to reflect the non-dimensional flow rate in each channel.
